# Integrated traditional Chinese medicine intervention for delaying HIV morbidity: study protocol for a multicentre randomised controlled trial

**DOI:** 10.1186/s13063-022-06625-x

**Published:** 2022-08-17

**Authors:** Xue Ding, Pengfei Meng, Xiuxia Ma, Weifeng Cui, Liangping Li, Xiyuan Song, Jiangrong Wang, Chengjie Ma, Xin Chen, Liran Xu

**Affiliations:** 1 The First Affiliated Hospital of Henan University of Chinese Medicine, 19 Renmin Road, Zhengzhou, 450099 China; 2 The Affiliated Hospital of Henan Academy of Chinese Medicine, Zhengzhou, 450004 China; 3 Shanghai Public Health Clinical Centre, Shanghai, 200000 China; 4 Beijing Ditan Hospital Capital Medical University, Beijing, 100000 China; 5 Kunming Municipal Hospital of Traditional Chinese Medicine, Kunming, 650000 China

**Keywords:** HIV, AIDS, Complementary medicine, Clinical trials

## Abstract

**Background:**

Acquired immune deficiency syndrome is caused by humans and is high worldwide. Active antiretroviral therapy emerged in the late 1990s and is effective against AIDS. However, despite the extensive research on AIDS, there is still no vaccine or cure. The benefits of traditional Chinese medicine (TCM) for AIDS are increasingly recognised, especially by patients with asymptomatic HIV infection.

**Methods/design:**

The proposed trial will enrol 216 eligible patients who will be randomised into treatment and control groups. After 72 weeks of intervention, the efficacy and safety of TCM for patients with AIDS will be assessed. The variables that will be measured include clinical symptoms, TCM syndromes, viral load, immunological indicators, inflammatory factors, quality of life, patient-reported outcomes and safety assessment.

**Discussion:**

The study aim to compare the effectiveness and safety of TCM for asymptomatic AIDS and explore its potential underlying mechanism. Additionally, the findings will provide a reference for the use of TCM to delay the onset and control the progression of HIV/AIDS.

**Trial registration:**

Chinese Clinical Trial Registry ChiCTR1800018365. Registered on 13 September 2018

## Background

Approximately 38 million people worldwide have human immunodeficiency virus (HIV), and 1.7 million people are newly infected each year [1]. The acquired immune deficiency syndrome (AIDS) pandemic is the most rigorous challenge for global public health [2]. Antiretroviral therapy (ART) is the most effective treatment for HIV/AIDS. However, ART is often limited by drug toxicity, poor therapeutic tolerance and drug resistance [3]. Therefore, it is necessary to identify new drugs or treatments. One such potential treatment is complementary and alternative medicine, which has been used worldwide to treat AIDS and its complications and includes therapies such as traditional Chinese medicine (TCM), acupuncture and Qigong. Several studies have shown that TCM can reduce drug side effects and mortality and improve immunity, clinical symptoms and quality of life [4, 5]. There is evidence that TCM is effective and safe for the treatment of asymptomatic AIDS [6–10]. However, there is insufficient evidence for the efficacy of TCM for patients with AIDS. Hence, a rigorously designed, large-scale, multicentre, randomised trial is needed to assess the effectiveness of TCM for AIDS. We aim to conduct a multicentre, randomised, double-blind, placebo-controlled trial to evaluate the efficacy and safety of TCM for AIDS. The trial will investigate whether TCM is an effective treatment for patients with AIDS and evaluate the clinical efficacy of TCM in reducing AIDS morbidity. In this trial, the time and incidence of endpoint events will be the primary evaluation indices, and clinical symptoms, TCM syndromes, viral load, immunological indicators, inflammatory factors, quality of life, patient-reported outcomes and safety assessment as the secondary effect indices.

## Methods/design

### Study design

**Fig. 1:**
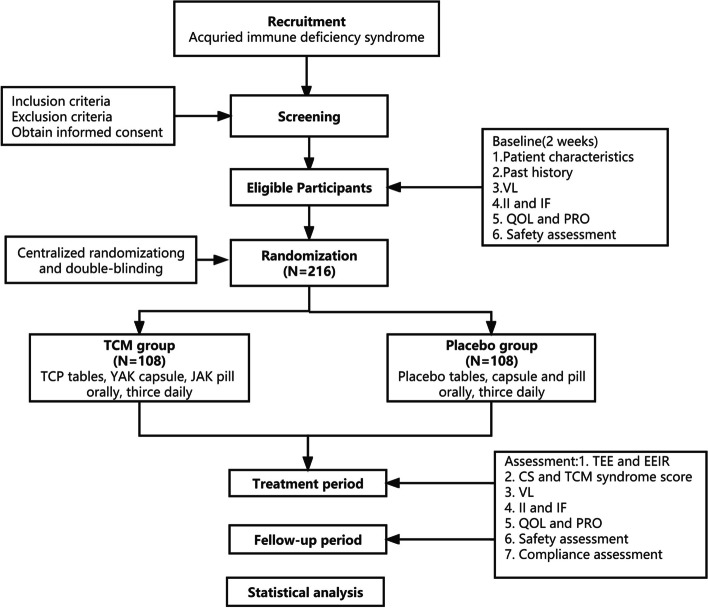
Flow of patients through the trial. TEE, time to endpoint events; EEIR, endpoint event incidence rate; CS, common symptom; VL, virological load; II, immunological indicator; IF, inflammatory factor; QOL, quality of life; PRO, patient-reported outcomes. Safety assessment: blood, urine, stool routine, liver and kidney function, electrocardiogram and chest X-ray. YAK, Yiaikang capsule; TCP, Tangcaopian tables; JAK, Jianaikang pill

This will be a multicentre, randomised, double-blind, simulated, parallel-controlled clinical trial. Participants will be recruited from six centres: Shanghai Public Health Clinical Centre; Beijing Ditan Hospital, Capital Medical University; Sichuan Academy of Chinese Medical Sciences; Ruikang Hospital Affiliated to Guangxi University of Chinese Medicine; Kunming Municipal Hospital of Traditional Chinese Medicine; and the First Affiliated Hospital of Henan University of Chinese Medicine. The study schema and estimated recruitment numbers are presented in Fig. 1. Eligible participants will be randomised to be assigned to the test group (TCM groups) or control groups (placebo) in a 1:1 ratio. Both groups will undergo a treatment period of 72 weeks and a follow-up period. Nineteen visits will be scheduled for each patient: baseline and each 4 weeks, 4, 8, 12, 16, 20, 24, 28, 32, 36, 40, 44, 48, 52, 56, 60, 64, 68 and 72.

### Ethics and communication

The study received ethical approval from the Research Ethics Committee of the First Affiliated Hospital of Henan University of Chinese Medicine (Approval No. 2018HL-042). This trial adhered to the Declaration of Helsinki and has been registered at the Chinese Clinical Trials Registry (ChiCTR1800018365) on September 13, 2018. Patients willing to participate will sign a consent form prior to participating.

### Setting and participants

Participants will be recruited from six large comprehensive hospitals in China: (1) Shanghai Public Health Clinical Centre; (2) Beijing Ditan Hospital, Capital Medical University; (3) Sichuan Academy of Chinese Medical Sciences; (4) Ruikang Hospital Affiliated to Guangxi University of Chinese Medicine: (5) Kunming Municipal Hospital of Traditional Chinese Medicine; and (6) the First Affiliated Hospital of Henan University of Chinese Medicine.

Inclusion criteria are as follows:

1. Patients who meet the criteria of HIV/AIDS diagnostic and chosen not to use ART
2. Number of CD4+ T cells ranges from 250 to 500/μL
3. Patients whose symptoms meet the Qi deficiency, damp heat and spleen and kidney deficiency according to the ‘Diagnostic criteria for HIV/AIDS (WS293-2008)’, ‘Clinic terminology of traditional Chinese medical diagnosis and treatment syndromes (2002)’ [11] and the ‘TCM diagnosis and treatment criteria for HIV/AIDS (2016)’ [12]
4. Patients aged 18–65 years and of either sex
5. Agree to sign the informed consent voluntarily

Exclusion criteria are as follows:

1. Patients who are participating in other clinical trials or have participated in other clinical trials within the last 3 months
2. Patients receiving highly active antiretroviral therapy
3. Patients with a combination of severe organic heart disease and severe arrhythmia, patients with abnormal liver function (alanine transaminase or aspartate transaminase greater than two times the upper limit of normal values), patients with abnormal renal function (serum creatinine greater than 1.5 times the upper limit of the normal value), patients with active tuberculosis or patients undergoing antituberculosis treatment and patients with haematologic disorders
4. Patients with combined tumours
5. Patients with severe psychiatric or neurological disorders
6. Pregnant or lactating women or women preparing for pregnancy
7. Patients with allergies and patients who are allergic to the test substances
8. Patients with a history of alcoholism (drinking more than 150 mL of alcohol per day) or those with manifestations of alcohol dependence syndrome

### Randomisation and blinding

Centralised randomisation was carried out by the Institute of Basic Research in Clinical Medicine, China Academy Of Chinese Medical Sciences, using the Clinical Research System (CRS), a remote electronic data capture. When an eligible participant is enrolled, the CRC has to register the detailed information of the participant on CRS. Then, after the confirmation of the investigator, the participant will be assigned to either the test or control groups in an allocation ratio of 1:1. The CRS will automatically generate the participant’s ID numbers and feedback to the investigator.

This trial is a double-blind trial in which the participants and investigators are blinded. The patients will receive a TCM drug or placebo.

### Interventions

Eligible patients will be randomised into two groups: receiving a placebo or a TCM treatment. The TCM regimens include three kinds of TCM treatment corresponding to the three most common types of TCM syndromes in HIV/AIDS patients. As shown in Table 1, participants with indications of a syndrome type, including all clinical symptoms, tongue and pulse, and more than two systemic symptoms, will be diagnosed with the type by TCM physicians and received the matched drugs. Since participants’ symptoms are likely to change over time along with the treatment, the diagnosis of types will be redone before each cycle to determine which kind of CMP is the most tailored one.

**Table 1:** Indication of TCM and placebo

Name of CMP	Syndrome types	Indications		
		Primary symptoms	Second symptoms	Tongue and pulse
YAK capsule Placebo YAK capsule	Qi deficiency syndrome	Fatigue, fatigue, lazy words	Dizziness, pale complexion, palpitations, spontaneous sweating	Slight light or normal tongue, weak or normal pulse
TCP tablets Placebo TCP tablets	Stagnant and congested damp-heat syndrome	Heavy-headedness, fatigue	Chest tightness, gastric distention, mouth stick, anorexia inappetence, loose stool, women with leucorrhea sticky and smelly	Red tongue with thick and greasy or yellow greasy coating, moistening or sliding pulse
JPYQ pills Placebo JPYQ pills	Spleen-kidney deficiency syndrome	Fatigue, lumbar and knee tenderness or lumbago	Abdominal distention, loss of appetite, chill, pale complexion, loose stool, frequent of urination, tinnitus	Light or thin tongue with white or slippery, sunken and wiry pulse

The diagnosis criteria of a syndrome type are that the participant has one of the primary symptoms, supporting evidence of tongue and pulse and more than 2-s symptoms. The corresponding CMP will be used in accordance with syndrome types and randomisation

*Abbreviations*: *CMP* Chinese medicine preparation, *YAK* Yi Ai Kang, *TCP* Tang Cao Pian, *JPYQ* Jian Pi Yi Qi pills

For the TCM group, patients will be given TCM interventions based on the TCM syndrome patterns, respectively, which are TCP tablets for stagnant and congested damp heat syndrome, YAK capsules for Qi deficiency and JAK pills for spleen-kidney deficiency syndrome. The TCM interventions are also recognised as a whole comprehensive intervention. The TCM group is component preparations of Chinese herbs, and its main components are shown in Table 2. YAK capsules (0.5 g per softgel) will give five softgel each time, TCP tablets (0.5 g per tablet) will give eight tablets each time and JAK pills (5 g per bag) will give one bag each time. All types of drugs will be given orally, thrice a day for 72 weeks. TCP tablets and placebo are produced by Shanghai Hundreds Ace Herbal Pharmaceutical Co., Ltd., Shanghai, China. YAK capsules, JAK pills and each placebo are made by the Affiliated Hospital of Henan Academy of Chinese Medicine, Zhengzhou, China.

**Table 2:** Main components of traditional Chinese medicine treatment

Chinese name	Latin name
**TCPb tables for stagnant and congested damp-heat syndrome**	
Huangqi	Astragali Radix
Laoguancao	Geranium Wilfordii Maxim.
Mumianhua	Gossampiniflos
Nuodaogen	Oryzae Glutinosae Radix
**YAK capsules for Qi deficiency**	
Renshen	Panax Ginseng C. A. Mey.
Baizhu	Atractylodes Macrocephala Koidz.
Fuling	Poria Cocos (Schw.) Wolf.
Dadouhuangjuan	Semen Sojae Germinatum
**JPYQ pills for spleen-kidney deficiency syndrome**	
Hongjingtian	Panax Ginseng C. A. Mey.
Gouqizi	Lycii Fructus
Lingzhi	Ganoderma
Renshen	Panax Ginseng C. A. Mey.

While those in the control group will take placebo granules composed of 90% and Herba pogostemonis (10%) in order to achieve the same colour, smell, taste and texture, and the participants are required to return the drug boxes and labels to the clinical research coordinator (CRC) as evidence of timely medication.

### Data collection

To maintain the quality of the research, researchers in each centre will be trained before the clinical trial. We will collect basic data from each patient including their characteristics and past history of HIV/AIDS, outcomes and safety assessment data at baseline and follow them up until the end of the trial (Table 3). After review by clinical inspectors, completed CRFs, data entry and management are completed by two individual data administrators to ensure the accuracy of the data.

**Table 3:** Schedule of data collection

	Screening and baseline	Treatment period														Follow-up period			
Visits	1	2	3	4	5	6	7	8	9	10	11	12	13	14	15	16	17	18	19
Week number	− 7 to 0	4	8	12	16	20	24	28	32	36	40	44	48	52	56	60	64	68	72
Time window (days)	×	± 6	± 6	± 6	± 6	± 6	± 6	± 6	± 6	± 6	± 6	± 6	± 6	± 6	± 6	± 6	± 6	± 6	± 6
Inclusion/exclusion criteria	×																		
Informed consent	×																		
History/demographics	×																		
Time to endpoint events or endpoint incidence		×	×	×	×	×	×	×	×	×	×	×	×	×	×	×	×	×	×
Common symptom score	×	×	×	×	×	×	×	×	×	×	×	×	×	×	×	×	×	×	×
TCM syndrome score	×	×	×	×	×	×	×	×	×	×	×	×	×	×	×	×	×	×	×
VL	×																		×
CD4+, CD8, CD4/CD8	×			×			×			×			×			×			×
NK, CD4+CD45RA+T, CD4+CD45RO+T	×																		×
IL-4, IL-17, TNF-a, INF-γ	×																		×
QOL and PRO	×						×						×						×
Blood, urine, stool routine	×						×						×						×
Liver and kidney function	×						×						×						×
Electrocardiogram	×						×						×						×
Physical examination	×						×						×						×
Adverse events	×	×	×	×	×	×	×	×	×	×	×	×	×	×	×	×	×	×	×
Combined medication	×	×	×	×	×	×	×	×	×	×	×	×	×	×	×	×	×	×	×

*Abbreviations*: *TCM* traditional Chinese medicine, *VL HIV* virological load, *NK* natural killer cell, *IL* interleukin, *QOL* quality of life, *PRO* patient-reported outcomes

### Assessment

#### Primary outcome measure

The primary outcome is the time to endpoint events (TEE) and endpoint event incidence (EEI) as the primary evaluation indicator. Endpoint event criteria will be defined by referring to the ‘Chinese guidelines for the diagnosis and treatment of AIDS (2018)’ [13].

#### Secondary outcomes

The secondary outcomes are as follows:

- Common symptom and TCM syndrome score: The effectiveness of TCM is evaluated using the common symptom sore method (Table 4) and the TCM syndrome sore method (Table 5). According to Guiding principles for clinical research of new drugs of TCM (Trial) (2002) [14], a reduction in TCM syndrome score by ≥ 30% indicates that the clinical symptoms improved or disappeared and the treatment is considered clinically effective. The TCM syndrome score is calculated as follows: [(scores before treatment − scores after treatment)÷scores before treatment] × 100%.
- Virological load: HIV virological load (VL) will be done at baseline and week 72 during the follow-up. According to and TCM diagnosis and treatment criteria for HIV/AIDS [15], ratings are effective, invalid and steady.
- Immunological indicators and inflammatory factors: CD4+ cell count, CD8+ cell count and CD4/CD4 will be done at baseline and weeks 12, 24, 48, 60 and 72 during the treatment period. According to and TCM diagnosis and treatment criteria for HIV/AIDS [15], ratings are effective, invalid and steady. Changes in inflammatory factors (IF) include interleukin(IL)-17, IL-4, tumour necrosis factor (TNF)-a, interferon-γ (INF-γ) and immunological indicator(II) including natural killer cell (NK), CD4+CD45RA+T lymphocytes, and CD4+CD45RO+T lymphocytes will be done at weeks 0 and 72 of the follow-up phase.
- Quality of life (QOL) and patient-reported outcomes (PRO): Assessment of the quality of life of PHIV by using the WHOQOL-HIV-brief [16]  and Patient Peport Outcomes Assessment Scale (PLWHA-PRO) [17]. They will be observed and recorded prior to treatment, at baseline and weeks 24, 48 and 72 of the follow-up phase.

**Table 4:** Evaluation criteria of common symptoms

Symptoms	Score		
	Mild = 3	Moderate = 6	Severe = 9
Fever	T ≤ 38.0 °C	38.0 °C < T < 38.5 °C	T ≥ 38.5 °C
Cough	Intermittent cough during the day	Intermittent cough during the day and occasionally at night	Frequent cough day and night
Expectoration	Expectoration 10-50 ml or expectoration 5-25 ml at night and morning	Expectoration 50-100 ml or expectoration 5-25 ml at night and morning	Expectoration > 100 ml or expectoration 50-100 ml at night and morning
Weight loss	Weight loss ≤ 10%	10% < weight loss < 20%	Weight loss ≥ 20%
Cold	≤ 2 times/month or 1 time/2 weeks	3-4 times/month or 1 time/week	≥ 5 times/month or > 1 time/week
Rash	It occurred in some areas and lasted for a short time.	It occurred in many places < 1 month.	It occurred in the whole body extensively > 1 month.
Alopecia	Light alopecia	Moderate alopecia	Severe alopecia
Herpes	It occurred in some areas and lasted for a short time.	It occurred in many places < 1 month.	It occurs repeatedly, and the pain is unbearable.
Mucosal ulcer	< 2 small mucosal ulcers	3-5 mucosal ulcers	> 6 mucosal ulcers or broad area of mucosal ulcer

No symptom = 0

**Table 5:** Evaluation criteria of TCM syndrome score

Syndrome	Score		
	Mild = 3	Moderate = 6	Severe = 9
Fatigue	Slight fatigue can engage in physical labour.	Moderate fatigue will reluctantly engage in physical labour if insisted to do so.	Weak limbs, unable to do physical labour
Depression	Depressed, reluctant to speak	Depressed, drowsy, reluctant to speak	Extremely tired, hesitant to speak
Giddy	Occasional dizzy	Dizzy, unable to walk	Dizzy and fall down
Palpitation	Occasional palpitations	Occasional palpitations, lasting for a long time	Frequent palpitations, difficulty to calm down, and even affect daily life
Lustreless complexion	Pale complexion	Pale and bloodless complexion	Pale or yellow complexion
Spontaneous sweating	Less sweating	Moderate sweating	Severe sweating
Dizzy	Slight dizziness	Moderate dizziness	Severe dizziness
Body heavy	Light body heavy, freedom of activity	Moderate body heavy, reduced activity	Severe body heavy, no activity
Chest distress	Occasional chest distress	Frequent chest tightness	Persistent chest distress
Gastric distention	Slight gastric distention without affecting diet	Moderate gastric distention, diet reduction	Severe gastric distention, abstaining diet
Sticky mouth	Sticky mouth, without affecting diet	Moderate sticky mouth distention, diet reduction	Severe sticky mouth distention, abstaining diet
Anorexia	Loss of appetite	Appetite reduction	Anorexia
Loose stool	Loose stool, 1-2 times/day	Loose stool, 3-4 times/day	Loose stool, > 4 times/day
Abdominal distention	Slightly distended abdomen	Moderately distended abdomen	Severe distended abdomen
Aching of waist or knees	Slight ache of waist and knees	Moderate ache of the waist and knees	Severe ache of the waist and knees
Chilly limbs	The extremities are slightly cold	Cold limbs need to add clothing	Chills all over the body cannot be relieved by adding clothes
Frequency urination	Frequency of urination < 10 times/day	Frequency of urination 10-15 times/day	Frequency of urination > 15 times/day
Tinnitus	Mild of tinnitus	Tinnitus and difficulty hearing	Tinnitus and hearing impairment

No symptom = 0

### Safety assessments

The participants’ blood, urine, stool routine, liver and kidney function, electrocardiogram and chest X-ray will be monitored at baseline and weeks 24, 48 and 72 during the treatment period.

### Adverse event and oversight

Investigators will ask every subject at each visit whether they have experienced any adverse events (AEs) during the study period. If they have any AEs, the investigator will provide appropriate treatment to the subject immediately and record the AEs. In the case of serious adverse events (SAEs), the investigator will offer appropriate treatment to the subject immediately and report the event to the First Affiliated Hospital of Henan University of Chinese Medicine within 24 h from the time of recognition. If necessary, blinding will be broken by adequate procedure, and the documented procedure will be kept in the investigator’s study file. Any adverse events during the trial will be recorded in detail.

The oversight is in charge of Guangzhou Boji Contract Research Organization (CRO), through quarterly independently monitoring by clinical research associates (CRA) in terms of safety, quality and progress of the study.

### Sample size

A pilot experiment we performed showed that the incidence of endpoint events in the control group was 35%, and the incidence of endpoint events in the TCM group was 8%. Therefore, the required sample size is 33 cases in the control group and 66 cases in the treatment group. According to the preliminary project research and considering the typical 10% dropout rate of patients who participate in therapy, the final required sample size for the trial is 216 cases.

### Statistical analysis

All statistical analyses will be performed using SAS 9.0. All statistical tests will be two-sided, and *P* < 0.05 will be considered statistically significant. Baseline information, such as mean, standard deviation and minimum and maximum values, will be calculated to describe the quantitative indicators. Categorical indicators will be described by frequency. For the analysis of efficacy and safety indicators, Cox survival analysis will be used to compare the time to endpoint events between the two groups, and TCM symptoms will be analysed using the chi-square test or rank-sum test.

## Discussion

Patients with AIDS remain a major public health problem. TCM as a system of medicine has been a form of health care in China for several thousand years. Many studies show that TCM can reduce anti-retroviral side effects, increase patients’ immunity, improve QOL and clinical symptoms and prolong patients’ life and is an effective and safe TCM in treating HIV/AIDS [4–7]. However, the lack of high-quality research has hindered the development of evidence-based clinical research on HIV/AIDS and prompted the design of this clinical trial.

This trial has the following advantages: (1) This study is a randomised, double-blind, placebo-controlled, multicentre study that is scientific and rigorous. (2) The trial design is guided by the TCM theory of clinical treatment, which is based on symptom differentiation. (3) The study participants will be selected from a region of China that has a high prevalence of HIV infection, and the long-term follow-up will ensure the compliance of the participants.

The results of this clinical trial would reveal whether TCM can supplement Western medical treatment for AIDS. If the trial is successful, it will provide a new option for patients and physicians to reduce the cost and burden of AIDS care.

## Trial status

Patient recruitment started on 1 March 2020 and will be completed on 20 December 2021. Version number:2.0.

## Data Availability

The data that support the findings of this study are openly available in the “clinical evaluation centre of the Chinese Academy of Traditional Chinese Medicine”. Data or codes generated or used during the study are available from the corresponding author by request.

## Abbreviations

HIV: Human immunodeficiency virus
AIDS: Acquired immune deficiency syndrome
ART: Active antiretroviral therapy
TCM: Traditional Chinese medicine
CRC: Clinical research coordinator
CRS: Clinical research system
TEE: Time to endpoint events
EEI: Endpoint event incidence
VL: Virological load
IF: Inflammatory factors
IL: Interleukin
INF-γ: Interferon-γ
II: Immunological indicator
NK: Natural killer cell
QOL: Quality of life
PRO: Patient-reported outcomes
AEs: Adverse events
SAEs: Serious adverse events
CRO: Contract Research Organization
CRA: Clinical research associates
YAK: Yi Ai kang capsule
TCP: Tang Cao Pian tablets
JAK: Jian Ai Kang
PCP: Pneumocystis pneumonia
